# Research progress in endometriosis-associated ovarian cancer

**DOI:** 10.3389/fonc.2024.1381244

**Published:** 2024-04-25

**Authors:** Ling Tang, Ce Bian

**Affiliations:** Department of Gynecology and Obstetrics, Key Laboratory of Birth Defects and Related Diseases of Women and Children, Ministry of Education, West China Second Hospital, Sichuan University, Chengdu, Sichuan Province, China

**Keywords:** endometriosis, ovarian neoplasms, endometriosis-associated ovarian cancer, risk factors, diagnosis

## Abstract

Endometriosis-associated ovarian cancer (EAOC) is a unique subtype of ovarian malignant tumor originating from endometriosis (EMS) malignant transformation, which has gradually become one of the hot topics in clinical and basic research in recent years. According to clinicopathological and epidemiological findings, precancerous lesions of ovarian clear cell carcinoma (OCCC) and ovarian endometrioid carcinoma (OEC) are considered as EMS. Given the large number of patients with endometriosis and its long time window for malignant transformation, sufficient attention should be paid to EAOC. At present, the pathogenesis of EAOC has not been clarified, no reliable biomarkers have been found in the diagnosis, and there is still a lack of basis and targets for stratified management and precise treatment in the treatment. At the same time, due to the long medical history of patients, the fast growth rate of cancer cells, and the possibility of eliminating the earliest endometriosis-associated ovarian cancer, it is difficult to find the corresponding histological evidence. As a result, few patients are finally diagnosed with EAOC, which increases the difficulty of in-depth study of EAOC. This article reviews the epidemiology, pathogenesis, risk factors, clinical diagnosis, new treatment strategies and prognosis of endometriosis-associated ovarian cancer, and prospects the future direction of basic research and clinical transformation, in order to achieve stratified management and personalized treatment of ovarian cancer patients.

## 1 Introduction

Endometriosis (EMS) is a prevalent condition that significantly impacts the quality of life and reproductive function in women. According to statistics, the prevalence of EMS among women of childbearing age ranges from 5% to 10% (1), while it can reach as high as 20% to 60% in women experiencing pelvic pain or infertility (2). Despite its benign nature, EMS shares biological characteristics with malignant tumors, showing invasive, adhesive, and metastatic potentials, with a risk of malignant transformation. As epidemiological and molecular genetic research continues to reveal, EMS is closely related to epithelial ovarian cancer (EOC), especially ovarian clear cell carcinoma (OCCC) and ovarian endometrioid carcinoma (OEC). Therefore, ovarian cancers closely associated with endometriosis, which may arise malignantly from endometriosis, predominantly manifest as OCCC and OEC. Collectively, these are referred to as Endometriosis-associated ovarian cancer (EAOC). Early in 1925, Sampson (3) pioneered the demonstration of the correlation between EMS and ovarian cancer and subsequently proposed the pathological diagnostic criteria for EAOC. These criteria comprise: 1) the existence of cancerous tissue in proximity to endometriotic lesions, 2) exclusion of metastasis from other tumor sources, and 3) the presence of characteristic glandular epithelium surrounding endometriotic lesions. In 1953, Scott introduced an additional criterion (4): microscopic evidence of the transformation from endometriotic lesions to malignant tissue. Compared to non-EAOC patients, those with EAOC exhibit a younger age at diagnosis, an earlier onset of the disease, lower tumor grades, and lower recurrence rates (5), suggesting that EAOC represents a distinct subtype of solid tumors. At present, the diagnosis of EAOC mainly depends on surgery and pathological examination, but the rate of missed diagnosis is often increased due to the “burnout effect” of the tumor and the doctor’s neglect of EMS lesions when reading the film. By comprehensively reviewing the epidemiology, pathogenesis, risk factors, clinical diagnosis, treatment modalities and prognosis of EAOC, this review aims to elucidate the distinctive characteristics of EAOC, facilitate early identification by clinicians and provide a valuable reference for enhancing the prognostic outcomes associated with EAOC.

## 2 Epidemiology of EAOC

In the investigation, we have noted a relatively low risk of ovarian cancer in the general population, standing at merely 1.31% (6). Nevertheless, for individuals affected by EMS, the risk of ovarian cancer undergoes a significant escalation, exhibiting a relative risk of 2.51-fold (7), with a lifetime risk reaching 2.5% (8). Despite the comparatively modest overall incidence risk, the heightened attention is warranted due to the elevated mortality rate of ovarian cancer within gynecological cancers and the prevalent and chronic nature of EMS. In recent years, substantial interest has been directed towards researching whether individuals with endometriosis face an elevated risk of cancer. Consistent findings in the research field underscore that EMS significantly elevates the risk of OCCC and OEC. A study in the Netherlands involving 131,450 patients with histologically confirmed cases of endometriosis revealed incidence rate ratios for OCCC and for OEC (9) with similar incidence rates from a Chinese study (10).

## 3 EAOC pathogenesis

### 3.1 Molecular biology

Currently, high mutation frequencies are observed in the genes ARID1A, phosphatase and tensin homolog (PTEN), and phosphatidylinositol-4,5-bisphosphate 3-kinase catalytic subunit alpha (PIK3C) in EAOC (11). The ARID1A gene, encoding a crucial component of the SWI/SNF complex, is considered a tumor suppressor gene and is frequently mutated in various cancers, with the highest mutation rates found in the two ovarian cancers associated with endometriosis (12). By using gene sequencing technology, ARID1A mutations were identified in 46% of 55 cases of OCCC, 30% of 10 cases of OEC, and none of the 76 cases of high-grade serous ovarian carcinoma (13). PTEN, located on chromosome 10, is a tumor suppressor gene involved in cell regulation,inhibiting tumor cell proliferation, adhesion, metastasis, and angiogenesis (14, 15). The PI3K/AKT/mTOR (PI3K) pathway is a classical signaling pathway that plays a crucial role in regulating cell survival, growth, and proliferation, and mutations in this pathway are common in human cancers (16).

Previous research has indicated that ARID1A gene mutations in OCCC may be associated with the abnormal activation of the PI3K-AKT pathway (17), a key player in altering tumor growth, proliferation, and metastasis. This abnormal activation enhances the invasiveness of tumors, shortening the time to cancer recurrence and death, suggesting an unfavorable prognosis (18). However, a mouse experiment revealed that the sole loss of ARID1A gene function does not induce ovarian cancer. Deleting the ARID1A gene alone does not induce ovarian cancer in mice, but when the ARID1A and PTEN genes are simultaneously knocked out, 60% of mice develop ovarian cancer with intra-abdominal dissemination, and 40% exhibit excessive proliferation of ovarian epithelium (19). Further research by Chandler et al. indicated that simultaneous deletion of the ARID1A gene and activation of the PIK3CA gene can induce OCCC in mice (20). In addition, ARID1A mutation can lead to impaired interferon (IFN) gene expression and reduce tumor response to immunotherapy (21).

A recent study involving 1,623 EAOC patients, including 1,078 cases of OEC and 545 cases of OCCC, confirmed these findings (22). Specifically, the relationship between ARID1A loss/mutation, clinical characteristics, outcomes, CD8+ tumor-infiltrating lymphocytes (CD8+TIL), and DNA mismatch repair deficiency (MMRD) revealed ARID1A gene inactivation in 42% of OCCC and 25% of OEC. However, ARID1A inactivation did not significantly impact the overall survival and progression-free survival of OCCC and OEC. Nonetheless, the continuous advancement in targeted therapeutic approaches, synthetic lethal strategies, and the investigation of the prognostic significance of ARID1A in immune modulation therapy is ongoing, indicating potential implications for prognosis (23, 24). Additional genes associated with EMS malignancy and EAOC: tumor suppressor gene p53, hepatocyte nuclear factor 1 homeobox B (HNF-1β), β-catenin gene (CTNNB1), kirsten rat sarcoma viral oncogene (KRAS), protein kinase B (KT), MicroRNA (miRN) are detailed in **Table 1**.

**Table 1:** Genes associated with EAOC formation.

Genes	Current research
ARID1A	The mutation rate of ARID1A gene in OCCC was 42% and in OEC was 25% (22); Mutations activation the PI3K-AKT pathway, induction of tumorigenesis and allows tumor cell proliferation (17, 18); ARID1A interacted with Enhancer of Zeste 2 Polycomb Repressive Complex 2 Subunit (EZH2) antagonized EZH2-mediated IFN responsiveness, shape cancer immune phenotype and immunotherapy (21)
PTEN	Mutation occurs in the early stage of tumorigenesis (25); Acts in concert with ARID1A to induction of tumorigenesis (20); Promoted metastasis and chemoresistance in ovarian cancer sell (14, 15);
PI3KCA	The mutation rate of PI3KCA gene in OCCC was 32% (26); Mutations may occur in late-stage OCCC (27);
HNF-1β	Mutations are common in OCCC, hypomethylation patterns are oncogenic (28);
CTNNB1	Mutations occurred only in OEC (29);
p53	High expression in benign endometriotic lesions next to the endometrioid or clear cell carcinoma (30, 31); Involved in tumorigenesis of malignancies (32);
KRAS	The mutation rate of KRAS gene in EAOC was 29% (33); allows tumor cell proliferation;
AKT	Activation PI3K/AKT pathway; involved in the occurrence and progression of ovarian cancer (16);
miRNA	Mirnas are involved in the regulation of angiogenesis in ovarian cancer (34); miRNA levels can predict the occurrence of early EAOC (35);

Past studies have indicated that the tumor microenvironment, particularly cancer-associated mesenchymal stem cells (CA-MSCs), plays a crucial role in the growth of ovarian cancer. Atiya et al.research report highlighted a subset of endometriosis-associated mesenchymal stem cells (enMSCs) in endometriosis (36), characterized by the loss of CD10 expression. This subset, by increasing the expression of iron export proteins, elevated intracellular iron levels in OCCC, thereby promoting OCCC growth and enhancing resistance to chemotherapy. Significantly, CD10-enMSCs also rendered OCCC more sensitive to iron apoptosis inducers and dihydroartemisinin (DH), offering a potential intervention pathway for future OCCC treatment.

Building upon current research, Wilczyński et al. proposed the hypothesis that endometriosis stem cells might be the primary targets for the carcinogenesis of EAOC (37). They delineated the process of transformation from endometriosis stem cells to cancer stem cells and the steps involved in the evolution from endometriosis to EAOC. However, more robust evidence is needed to thoroughly elucidate the exact carcinogenic mechanisms of EAOC.

### 3.2 Estrogen and epigenetics

EMS, being an estrogen-dependent disease, fosters the accumulation of estrogen in the local microenvironment. Estrogen plays a crucial role in the progression of endometrial lesions to atypical hyperplasia and even malignancy (38). Understanding the changes in estrogen signaling pathway will help to reveal the mechanism of estrogen involved in the malignant transformation of EMS. Andersen et al. analyzed estrogen regulatory genes and found that inactivation of estrogen receptor ERα, decreased progesterone receptor (PR) levels, and increased estrogen receptor ERβ may be the driving factors for EMS malignant transformation (39). This transition, accompanied by the overexpression of genes induced by estrogen receptor ERα, such as nuclear receptor interacting protein 1 (NRIP1) in EAOC, and the derepression of estrogen receptor ERα target genes, like FGF18, may promote the development of lesions towards EAOC. Wang et al.found that estrogen can influence gene methylation, and the estrogen-DNMT1 signaling pathway might induce high methylation of runt-related transcription factor 3 (RUNX3) (40), thereby promoting the malignant transformation of EMS. Several studies have identified common epigenetic features between EMS and ovarian malignancies (2, 41, 42) with epigenetic modifications in EAOC involving non-coding miRNA and histone modifications. Future research should focus on the interaction between hormonal regulation and inflammatory responses during the transformation process to gain a more comprehensive understanding of the mechanisms underlying the development of EMS into EAOC.

### 3.3 Iron related oxidative stress

**Figure 1:**
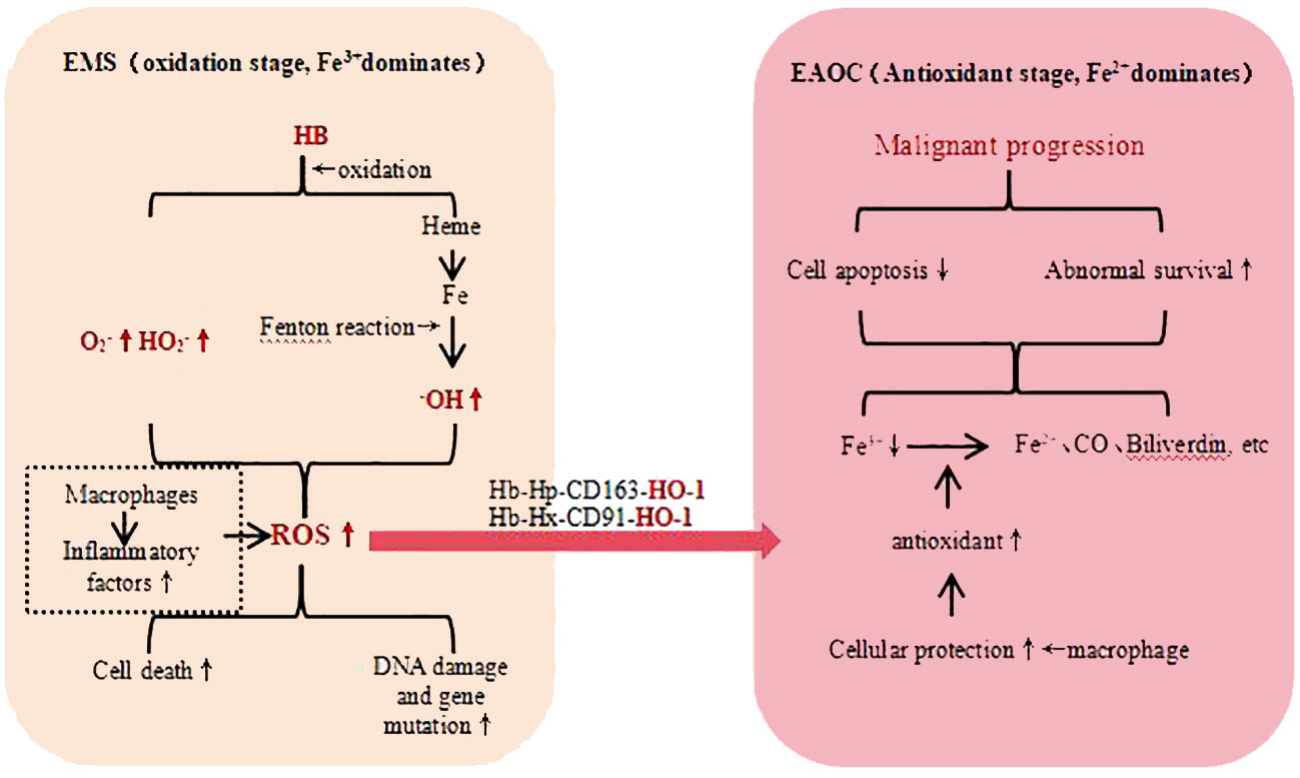
EMT Malignant Transformation - Iron Related Oxidative Stress. hemoglobin (HB), haptoglobin (HP), heme-binding glycoprotein (Hx), heme oxygenase-1 (OH-1), reactive oxygen species (ROS), carbon monoxide (CO), low-density lipoprotein receptor-related protein 1 (CD91), macrophage-specific protein (CD163), superoxide (O2^−^), perhydroxy (HO2^−^).

Elevated iron levels are considered a risk factor for cancer development, and patients with EMS often exhibit iron overload, which may be one of the factors contributing to EAOC. Iron is associated with cancer through a variety of mechanisms, including cancer metabolism, genome stability, and tumor microenvironment (43). Iron and its metabolites produce a large number of Reactive Oxygen Species (ROS) through Fenton reaction (44) and hemoglobin autooxidation (45), leading to DNA damage and acting as carcinogenic inducers in the process of EAOC. The body’s macrophage and other antioxidant defense systems are also activated, leading to the “two-step theory” of oxidative stress (**Figure 1**): The enhanced antioxidant capacity can protect cells from death or apoptosis, but at the same time, it also leads to DNA damage, genomic instability and mutation accumulation, thereby promoting the occurrence of tumors (46). In addition, iron-related oxidative stress can lead to the destruction of peritoneal mesothelial, which is conducive to the adhesion and metastasis of ectopic endometrial cells and tumor cells. Therefore, oxidative stress is a “double-edged sword” in the occurrence of EAOC (47).

### 3.4 Inflammatory response and immunodysregulation

EMS as a chronic inflammatory disease, creates a microenvironment in ovarian EMS that promotes inflammation, and sustained chronic inflammation may be a driving factor in inducing EAOC. Galectin, an important regulator of inflammation, shows high expression in EMS. Studies have found correlations between galectin-1, -3, and -9 and EAOC (48). In cancer cells, galectin is associated with the regulation of oncogenic signaling pathways, apoptosis, and changes in proliferation rates, making it a potential target for future cancer therapy (49).

The high-level expression of inflammasome complex genes (NLRP3, AIM2, PYCARD and NAIP) and inflammasome-related pathway genes (TLR1, TLR7, TOLLIP, NFKBIA and TNF) demonstrated their role in the progression of EMS and EAOC (50). However, there is still a lack of detailed analysis of the relevant immune components in the malignant transformation of EMS (51), and the exact immune pathways and cellular processes are still unclear, which is worthy of further research in the future.

## 4 EAOC risk factors

### 4.1 High estrogen state

A high estrogen state is considered a significant risk factor for the malignant transformation of EMS (52). Factors such as early menarche, infertility, or low parity keep patients in a prolonged state of endogenous high estrogen levels, increasing not only the likelihood of EMS but also the risk of EAOC. A stratified study on 66,450 women investigating 12 risk factors for epithelial ovarian cancer found that the risk of OEC gradually increases with earlier age at menarche and later age at menopause (53). Recent research exploring hormone replacement therapy (HRT) in postmenopausal women with a history of EMS found that, except for HRT using estrogen alone, other HRT regimens do not increase the risk of ovarian cancer in postmenopausal women with a history of endometriosis (54). This reflects the potential increased risk of EAOC with exogenous estrogen, highlighting different pathways in the role of endogenous and exogenous estrogen in the association between EMS and EAOC, deepening our understanding of this complex relationship.

### 4.2 Menopause

In a retrospective case-control study, Udomsinkul et al. identified menopause as a significant independent risk factor for EAOC (55). In postmenopausal women, ovarian function declines, leading to a significant decrease in estrogen levels. It is generally believed that postmenopausal patients may experience relief from symptoms of EMS due to the decline in estrogen levels. However, Giannella et al. reported an incidence of endometriosis in menopausal women to be 2-4% (56), highlighting the importance of special attention to this group. The decreased likelihood of physiological cysts and the increased risk of malignant transformation of ovarian masses in postmenopausal women make it a noteworthy consideration.

### 4.3 Age and the course of endometriosis

Current research indicates that age and the long-term development of EMS are important risk factors for EAOC patients. It is noteworthy that EAOC patients are diagnosed at a younger age, with the average diagnosis age being 48.65 years compared to 54.39 years for non-EAOC patients (57). In a study the longest duration of ovarian endometriotic cysts in EAOC patients was 23 years, with an average duration of 10 years (58). The study suggests that the long-term development of ovarian endometriotic cysts increases the risk of malignant transformation. Murakami et al.analyzed the medical history of EAOC patients and found that the median time from the diagnosis of endometriotic cysts to the diagnosis of EAOC was 36 months, with approximately 75% of patients progressing to EAOC within 60 months (59). Given the low incidence of EAOC, the phenomenon of endometriotic cysts rapidly progressing to cancer in a short period suggests that EAOC may occur in earlier, less detectable stages, highlighting the occult nature of EAOC and emphasizing the importance of identifying the risk in early-stage EAOC patients.

### 4.4 Hysterectomy

The relationship between hysterectomy and ovarian cancer is intricate. Previous studies suggest that hysterectomy may impede retrograde menstrual flow and the transfer of carcinogenic substances (60), thereby reducing the risk of ovarian cancer. Khoja et al. after accounting for confounding factors such as estrogen and estrogen-progestin use, as well as a history of EMS, found that the risk of ovarian cancer decreases only in women with a combination of hysterectomy and EMS (61), while there is no correlation in women without EMS. Ring et al. research also confirms that, although hysterectomy is not generally associated with the risk of ovarian epithelial cancer (62), it significantly reduces the risk of ovarian clear cell carcinoma.

In patients with endometriosis, the infrequent use of oral contraceptives, comorbid depression, or pelvic inflammation may elevate the risk of ovarian cancer (63). However, for patients with EAOC, there is currently a lack of well-designed studies providing conclusive evidence regarding these risk factors.

## 5 Clinical diagnosis of EAOC

### 5.1 Clinical symptoms and signs

Clinical symptoms and signs of EAOC are atypical, lacking specific diagnostic criteria. According to the “dualistic model of ovarian cancer”, researchers suggest that EAOC often belongs to Type I ovarian cancer, characterized by relative indolence, typically lower invasiveness, and less propensity for widespread dissemination (64). Symptoms of EAOC are often similar to those of endometriosis, mainly presenting as pelvic masses. Clinicians should be vigilant for EAOC when endometriosis patients exhibit typical cyclical pain rhythm changes, abnormal uterine bleeding, or if the mass has a maximum diameter >10 cm or shows rapid enlargement (65).

### 5.2 Tumor marker

Currently, there is a lack of specific and cost-effective biomarkers to identify the occurrence of EAOC. Serum carbohydrate antigen 125 (CA125) is the most commonly used ovarian tumor marker. Previous studies suggested that malignancy is likely when CA125 is >200 U/ml. However, CA125 is not highly specific, as it can be influenced by various factors such as endometriosis, inflammation, and menstruation. Its sensitivity in early-stage EAOC is also relatively low. In other study CA125 levels showed no significant statistical difference between patients with ovarian endometriotic cysts and those with EAOC (66).

Compared to CA125, carbohydrate antigen 19.9 (CA19.9) and human epididymal protein 4 (HE4) have advantages in diagnosing EAOC. CA19.9 is a potential serum marker for diagnosing EAOC; in Magalhães et al. study, a serum CA19.9 >22.31 U/ml showed a sensitivity of 82.14% in distinguishing between ovarian endometriotic cysts and EAOC (67). HE4, highly expressed in ovarian cancer and unaffected by endometriosis, exhibits high specificity. Xu et al. found that a serum HE4 >59.7 pmol/L could diagnose EAOC, with a specificity of 99.4% when HE4 >140 pmol/L (68). For epithelial ovarian cancer, the combined detection of HE4 and CA125 demonstrates higher sensitivity than CA125 alone. Multiple studies suggest that the joint examination of various tumor markers is more effective in diagnosing ovarian epithelial cancer (69). In a comprehensive review, concluded that the combination of CA125 and HE4 is currently the most effective diagnostic approach for ovarian epithelial cancer, but its discriminative ability for EAOC requires further clinical research and analysis for validation (70).

### 5.3 Radiology

Ultrasound plays a crucial role in the diagnosis of epithelial ovarian cancer. Typical features include cystic and solid masses, thick septa, associated solid nodules or papillary projections, and areas of necrosis. Ovarian cancer often presents with ascites and enlarged lymph nodes, with peritoneal, mesenteric, and omental metastases. In differentiating from EAOC, ultrasound examination should focus on specific characteristics of EAOC, such as a cystic lesion diameter larger than 10 cm or showing an increasing trend, having a unilocular or multilocular solid component, and rich blood flow signals (71). The disappearance of ground glass echoes is also indicative of malignancy (72). Moreover, EAOC typically manifests as a unilateral cystic lesion with papillary projections, and ascites is less commonly observed (73).

Magnetic Resonance Imaging (MRI) with its excellent soft tissue resolution and multi-planar imaging advantages offers greater accuracy in differentiating EAOC compared to Computerized Tomography (CT). A study found that Whole-Body Diffusion-Weighted Imaging/MRI (WB-DWI/MRI) achieved an accuracy of 93% in determining the benign or malignant nature of ovarian masses, significantly higher than CT’s accuracy of 82% (74). Using MRI relaxation method to measure the total iron concentration and transverse relaxation rate of cyst fluid in ovarian endometriosis cysts can predict the malignant transformation of ovarian endometriosis (75). In Zhang X et al. research, using MRI to depict the features of EAOC and non-EAOC, revealed that EAOC, especially clear cell ovarian cancer, more commonly presents as a unilocular cystic mass (76), showing statistically significant lateralization. Cystic fluid exhibits low signal intensity on T2-weighted imaging, and focal nodular growth patterns are more frequent. These findings underscore the critical role of ultrasound and MRI in the diagnosis of EAOC.

## 6 Progress in the management and treatment of EAOC

Most ovarian cancer patients experience recurrence within approximately three years. Advanced ovarian cancer and recurrent cases often exhibit resistance to platinum-based drugs, leading to a deterioration in clinical prognosis (77), making ovarian cancer treatment a longstanding challenge in gynecologic oncology. Compared to the common high-grade serous ovarian carcinoma, EAOC has a lower incidence rate, but it shows better early prognosis, although the late-stage survival rate is significantly lower than high-grade serous ovarian carcinoma. EAOC patients generally exhibit poorer response to platinum-based chemotherapy compared to non-EAOC cases (78, 79). Current experience in EAOC treatment primarily stems from studies on epithelial ovarian cancer. The initial standard treatment for EAOC includes surgery followed by platinum-based chemotherapy (77). Early-stage EAOC patients should undergo comprehensive staging surgery, while for intermediate to late-stage EAOC patients, consideration should be given to primary debulking surgery (PDS) upon preoperative or intraoperative assessment of extra-ovarian metastasis. Surgery should aim to remove all macroscopically visible tumors to reduce tumor burden, enhance chemotherapy efficacy, and improve prognosis.

### 6.1 Lymphadenectomy

Lymph nodes serve as crucial pathways for solid tumor metastasis. Systematic lymph node dissection in early-stage ovarian cancer patients is valuable for determining tumor staging, however it is not known whether it is beneficial for prognosis. EAOC as a specific subtype of ovarian epithelial cancer, is often diagnosed in its early stages. Recent evidence from a multicenter retrospective study suggests that early-stage and low-grade endometrioid ovarian cancer patients who undergo lymph node dissection have superior 5-year disease-free survival and overall survival rates compared to those who do not undergo lymph node dissection (80), with rates of 92.0% vs. 85.6% (*p*=0.016) and 97.7% vs. 92.8% (*p*=0.013), respectively. Another prospective, multicenter, randomized phase III clinical trial designed by Deng et al. in 2023 is ongoing. By comparing the progression-free survival (PFS) and overall survival (OS) outcomes of patients with stage IA-IIB epithelial ovarian cancer who undergo lymph node dissection surgery versus those who do not (81), this study aims to provide more precise evidence regarding the efficacy and safety of early lymph node surgery. The benefits and drawbacks of performing lymph node dissection in advanced ovarian cancer patients have been elucidated by high-quality evidence. A multicenter, phase III randomized controlled trial published in the New England Journal of Medicine in 2019 demonstrated that systematic pelvic and para-aortic lymph node dissection did not prolong patients’ OS or PFS and was associated with a higher incidence of postoperative complications (82). Subsequently, the National Comprehensive Cancer Network (NCCN) guidelines adjusted the indications for lymph node dissection surgery.

### 6.2 Intraperitoneal chemotherapy

A small proportion of EAOC is diagnosed in advanced stages, where achieving complete resection through surgery is challenging. Researchers have long attempted to enhance drug efficacy through intraperitoneal chemotherapy, particularly for advanced ovarian cancer. Early clinical trials conducted by the Gynecologic Oncology Group (GOG), including GOG-104, GOG-114, GOG-172, and GOG-252, failed to establish intraperitoneal chemotherapy as a first-line treatment due to design flaws, insufficient statistical evidence, and a higher likelihood of adverse reactions. Hyperthermic intraperitoneal chemotherapy (HIPEC), which combines thermal therapy and intraperitoneal perfusion treatment with intraperitoneal chemotherapy, has become a hot topic in debulking surgery for advanced ovarian cancer in recent years. In 2018, Van Driel et al. demonstrated that adding HIPEC to stage III epithelial ovarian cancer patients led to longer recurrence-free survival and overall survival without increasing the incidence of side effects (83). The clinical trial OVHIPEC-1 reported by Aronson et al. in 2023 confirmed a 10-year survival benefit of HIPEC in primary stage III epithelial ovarian cancer patients undergoing interval cytoreduction surgery (84). The efficacy of HIPEC in patients suitable for initial cytoreduction surgery remains uncertain. The OVHIPEC-2 trial, initiated in January 2020, is expected to provide results in this regard (85). However, the statistical results of the HIPECOVA trial conducted by Villarejo Campos et al. in 2024 failed to demonstrate a significant improvement in the prognosis of ovarian cancer patients with HIPEC (86). Therefore, HIPEC treatment remains experimental rather than standard therapy.

### 6.3 Drug chemotherapy

Currently, the standard first-line treatment regimen for EOC and EAOC is platinum-based combination chemotherapy, specifically carboplatin plus intravenous paclitaxel administered every 3 weeks for a total of 6 cycles. The JGOG 3016 trial previously reported significant improvements in progression-free survival and overall survival with a weekly dose-dense paclitaxel regimen and a 3-weekly carboplatin regimen, whereas the ICON8 trial did not observe this benefit. These trials have different strengths and weaknesses, and the differences may be related to pharmacogenomics or other factors such as dose intensity. The findings of Clamp et al. in 2022 confirmed that weekly dose-dense first-line chemotherapy did not improve overall survival or progression-free survival compared to standard 3-weekly chemotherapy (87). Therefore, the 3-weekly regimen chemotherapy remains the first-line approach.

Late-stage EAOC carries a poor prognosis, warranting in-depth research into targeted therapy and immunotherapy. Currently, molecular targeted therapies for ovarian cancer, such as poly ADP-ribose polymerase (PARP) inhibitors and the anti-angiogenic agent bevacizumab, have shown favorable outcomes in maintenance therapy for epithelial ovarian cancer patients with BRCA mutations, thereby extending the survival of ovarian cancer patients to some extent (88). However, even with satisfactory tumor reduction achieved through surgery and standardized chemotherapy and maintenance therapy, cancer patients may still experience treatment failure due to platinum resistance or tumor recurrence, highlighting the need to enhance drug efficacy and prolong recurrence-free survival. Mirvetuximab soravtansine (MIRV), an antibody-drug conjugate targeting folate receptor (FR) alpha, has shown promising efficacy when combined with bevacizumab in platinum-resistant recurrent ovarian cancer patients. Mirvetuximab soravtansine (MIRV) is a folate receptor (FR)-targeting antibody-drug conjugate (DC). In 2020, researchers found that MIRV combined with bevacizumab demonstrated good efficacy in treating platinum-resistant recurrent ovarian cancer patients. The confirmed objective response rate (ORR) was 39%, with a particularly effective response observed in the subset of platinum-resistant ovarian cancer patients with high FRα expression, achieving an ORR of 56%. The median duration of response was 12 months, and the PFS was 9.9 months (89). Phase 2 clinical studies of MIRV in epithelial ovarian cancer patients reported in 2023 further demonstrated its anti-tumor activity, along with good tolerability and safety, providing encouraging results (90). EAOC is highly likely to originate from endometriosis-associated ovarian cysts, which are often considered complex immune-related diseases. Immunotherapy has shown great potential in the treatment of EOC and EAOC. However, previous large phase III studies exploring the addition of immunotherapy to standard first-line treatment regimens have been disappointing, including the IMagyn050/GOG 3015/ENGOT-OV39 (91) and JAVELIN Ovarian 100 (92) studies. A turning point in immunotherapy emerged in 2023 with the release of interim data from the global multicenter phase III DUO-O study, showing promising clinical efficacy, warranting continued attention.

Recently, based on the establishment of animal models of endometriosis, successful reports of establishing EAOC mouse models have also emerged (93). By simulating tumor characteristics and reproducing the biological properties of tumors, these models can provide important reference for clinical precision treatment research, which is crucial for the study and development of precision treatment for EAOC.

## 7 Conclusion

The intricate relationship between EMS and ovarian cancer warrants in-depth investigation. Early identification of high-risk individuals for cancer among endometriosis patients is of paramount importance, necessitating the development of early detection methods and close monitoring. Future research directions in understanding the mechanisms and molecular genetics of EAOC may involve the utilization of advanced technologies, such as next-generation sequencing and whole transcriptome sequencing, as personalized diagnostic tools. The objective is to identify and confirm the driver mutations and candidate genes associated with the malignant transformation of EMS. These efforts hold the potential to provide more precise targeted therapies and immunotherapies for ovarian cancer, thereby improving patient prognosis and survival outcomes.

## Author contributions

LT: Writing – original draft. CB: Writing – review & editing.

## References

[1] Taylor HS Kotlyar AM Flores VA. Endometriosis is a chronic systemic disease: clinical challenges and novel innovations. Lancet. (2021) 397:839–52. doi: 10.1016/S0140-6736(21)00389-5 33640070

[2] Brunty S Mitchell B Bou-Zgheib N Santanam N. Endometriosis and ovarian cancer risk, an epigenetic connection. Ann Transl Med. (2020) 8:1715. doi: 10.21037/atm-20-2449 33490227 PMC7812227

[3] Sampson JA. Endometrial carcinoma of the ovary, arising in endometrial tissue in that organ. Arch Surg. (1925) 10:1–72. doi: 10.1001/archsurg.1925.01120100007001

[4] Scott R. Malignant change in endometriosis. Obstet Gynecol. (1953) 2:293–9. 13087921

[5] Bassiouny D El-Baz MA Gamil TM Shams N Ismiil N Dubé V. Endometriosis-associated ovarian cancer is a subset with a more favorable outcome and distinct clinical-pathologic characteristics. Int J Gynecol Pathol. (2019) 38:435–42. doi: 10.1097/PGP.0000000000000533 30059454

[6] Sorbi F Capezzuoli T Saso S Fambrini M Corda M Fantappiè G. The relation between endometrioma and ovarian cancer. Minerva Obstet Gynecol. (2021) 73:347–53. doi: 10.23736/S2724-606X.21.04757-2 34008389

[7] Eoh KJ Han M Kim EH Jung I Kim YT. Markedly increased risk of Malignancies in women with endometriosis. Gynecol Oncol. (2021) 161:291–6. doi: 10.1016/j.ygyno.2021.01.019 33514482

[8] Kvaskoff M Mahamat-Saleh Y Farland LV Shigesi N Terry KL Harris HR. Endometriosis and cancer: a systematic review and meta-analysis. Hum Reprod Update. (2021) 27:393–420. doi: 10.1093/humupd/dmaa045 33202017

[9] Hermens M van Altena AM Nieboer TE Schoot BC van Vliet HA Siebers AG. Incidence of endometrioid and clear-cell ovarian cancer in histological proven endometriosis: the ENOCA population-based cohort study. Am J Obstet Gynecol. (2020) 223:107.e1–107.e11. doi: 10.1016/j.ajog.2020.01.041 31981507

[10] He ZX Shi HH Fan QB Zhu L Leng J Sun D. Predictive factors of ovarian carcinoma for women with ovarian endometrioma aged 45 years and older in China. J Ovarian Res. (2017) 10:45. doi: 10.1186/s13048-017-0343-2 28716151 PMC5514486

[11] Murakami K Kotani Y Nakai H Matsumura N. Endometriosis-associated ovarian cancer: the origin and targeted therapy. Cancers (Basel). (2020) 12:1676. doi: 10.3390/cancers12061676 32599890 PMC7352633

[12] Mullen J Kato S Sicklick JK Kurzrock R. Targeting ARID1A mutations in cancer. Cancer Treat Rev. (2021) 100:102287. doi: 10.1016/j.ctrv.2021.102287 34619527

[13] Wiegand KC Shah SP Al-Agha OM Zhao Y Tse K Zeng T. ARID1A mutations in endometriosis-associated ovarian carcinomas. N Engl J Med. (2010) 363:1532–43. doi: 10.1056/NEJMoa1008433 PMC2976679 20942669

[14] Taylor H Laurence ADJ Uhlig HH. The role of PTEN in innate and adaptive immunity. Cold Spring Harb Perspect Med. (2019) 9:a036996. doi: 10.1101/cshperspect.a036996 31501268 PMC6886458

[15] Zheng F Zhong J Chen K Shi Y Wang F Wang S. PINK1-PTEN axis promotes metastasis and chemoresistance in ovarian cancer via non-canonical pathway. J Exp Clin Cancer Res. (2023) 42:295. doi: 10.1186/s13046-023-02823-w 37940999 PMC10633943

[16] Ediriweera MK Tennekoon KH Samarakoon SR. Role of the PI3K/AKT/mTOR signaling pathway in ovarian cancer: Biological and therapeutic significance. Semin Cancer Biol. (2019) 59:147–60. doi: 10.1016/j.semcancer.2019.05.012 31128298

[17] Huang HN Lin MC Huang WC Chiang YC Kuo KT. Loss of ARID1A expression and its relationship with PI3K-Akt pathway alterations and ZNF217 amplification in ovarian clear cell carcinoma. Mod Pathol. (2014) 27:983–90. doi: 10.1038/modpathol.2013.216 24336158

[18] Luchini C Veronese N Solmi M Cho H Kim JH Chou A. Prognostic role and implications of mutation status of tumor suppressor gene ARID1A in cancer: a systematic review and meta-analysis. Oncotarget. (2015) 6:39088–97. doi: 10.18632/oncotarget.5142 PMC4770758 26384299

[19] Cancer Genome Atlas Research Network Kandoth C Schultz N Kristian Cibulskis Eric Lander . Integrated genomic characterization of endometrial carcinoma [published correction appears in Nature. 2013 Aug 8;500(7461):242]. Nature. (2013) 497:67–73. doi: 10.1038/nature12113 23636398 PMC3704730

[20] Chandler RL Raab JR Vernon M Magnuson T Schisler JC. Global gene expression profiling of a mouse model of ovarian clear cell carcinoma caused by ARID1A and PIK3CA mutations implicates a role for inflammatory cytokine signaling. Genom Data. (2015) 5:329–32. doi: 10.1016/j.gdata.2015.06.027 PMC4583684 26484281

[21] Li J Wang W Zhang Y Cieślik M Guo J Tan M. Epigenetic driver mutations in ARID1A shape cancer immune phenotype and immunotherapy. J Clin Invest. (2020) 130:2712–26. doi: 10.1172/JCI134402 PMC7190935 32027624

[22] Heinze K Nazeran TM Lee S Krämer P Cairns ES Chiu DS. Validated biomarker assays confirm that ARID1A loss is confounded with MMR deficiency, CD8+ TIL infiltration, and provides no independent prognostic value in endometriosis-associated ovarian carcinomas. J Pathol. (2022) 256:388–401. doi: 10.1002/path.5849 34897700 PMC9544180

[23] Ogiwara H Takahashi K Sasaki M Kuroda T Yoshida H Watanabe R. Targeting the vulnerability of glutathione metabolism in ARID1A-deficient cancers. Cancer Cell. (2019) 35:177–190.e8. doi: 10.1016/j.ccell.2018.12.009 30686770

[24] Mandal J Mandal P Wang TL Shih IM. Treating ARID1A mutated cancers by harnessing synthetic lethality and DNA damage response. J BioMed Sci. (2022) 29:71. doi: 10.1186/s12929-022-00856-5 36123603 PMC9484255

[25] Martini M Ciccarone M Garganese G Maggiore C Evangelista A Rahimi S. Possible involvement of hMLH1, p16(INK4) and PTEN in the Malignant transformation of endometriosis. Int J Cancer. (2002) 102:398–406. doi: 10.1002/ijc.10715 12402310

[26] Zannoni GF Improta G Pettinato A Brunelli C Troncone G Scambia G. Molecular status of PI3KCA, KRAS and BRAF in ovarian clear cell carcinoma: an analysis of 63 patients. J Clin Pathol. (2016) 69:1088–92. doi: 10.1136/jclinpath-2016-203776 27153872

[27] Nakayama K Kyo S. PI3KCA in ovarian clear cell carcinoma-reply. Hum Pathol. (2014) 45:2515. doi: 10.1016/j.humpath.2014.07.024 25257578

[28] Chandra S Srinivasan S Batra J. Hepatocyte nuclear factor 1 beta: A perspective in cancer. Cancer Med. (2021) 10:1791–804. doi: 10.1002/cam4.3676 PMC7940219 33580750

[29] Nguyen VHL Hough R Bernaudo S Peng C. Wnt/β-catenin signalling in ovarian cancer: Insights into its hyperactivation and function in tumorigenesis. J Ovarian Res. (2019) 12:122. doi: 10.1186/s13048-019-0596-z 31829231 PMC6905042

[30] Nezhat F Cohen C Rahaman J Gretz H Cole P Kalir T. Comparative immunohistochemical studies of bcl-2 and p53 proteins in benign and Malignant ovarian endometriotic cysts. Cancer. (2002) 94:2935–40. doi: 10.1002/cncr.10566 12115382

[31] Silwal-Pandit L Langerød A Børresen-Dale AL. TP53 mutations in breast and ovarian cancer. Cold Spring Harb Perspect Med. (2017) 7:a026252. doi: 10.1101/cshperspect.a026252 27815305 PMC5204332

[32] Wang CK Chen TJ Tan GYT Chang FP Sridharan S Yu CHA. MEX3A mediates p53 degradation to suppress ferroptosis and facilitate ovarian cancer tumorigenesis. Cancer Res. (2023) 83:251–63. doi: 10.1158/0008-5472.CAN-22-1159 PMC9845988 36354374

[33] Stewart CJ Leung Y Walsh MD Walters RJ Young JP Buchanan DD. KRAS mutations in ovarian low-grade endometrioid adenocarcinoma: association with concurrent endometriosis. Hum Pathol. (2012) 43:1177–83. doi: 10.1016/j.humpath.2011.10.009 22305241

[34] Stieg DC Wang Y Liu LZ Jiang BH. ROS and miRNA dysregulation in ovarian cancer development, angiogenesis and therapeutic resistance. Int J Mol Sci. (2022) 23:6702. doi: 10.3390/ijms23126702 35743145 PMC9223852

[35] Gahlawat AW Witte T Haarhuis L Schott S. A novel circulating miRNA panel for non-invasive ovarian cancer diagnosis and prognosis. Br J Cancer. (2022) 127:1550–6. doi: 10.1038/s41416-022-01925-0 PMC9553930 35931806

[36] Atiya HI Frisbie L Goldfeld E Orellana T Donnellan N Modugno F. Endometriosis-associated mesenchymal stem cells support ovarian clear cell carcinoma through iron regulation. Cancer Res. (2022) 82:4680–93. doi: 10.1158/0008-5472.CAN-22-1294 PMC9755968 36219681

[37] Wilczyński JR Szubert M Paradowska E Wilczyński M. Endometriosis stem cells as a possible main target for carcinogenesis of endometriosis-associated ovarian cancer (EAOC). Cancers (Basel). (2022) 15:111. doi: 10.3390/cancers15010111 36612107 PMC9817684

[38] He J Chang W Feng C Cui M Xu T. Endometriosis Malignant transformation: epigenetics as a probable mechanism in ovarian tumorigenesis. Int J Genomics. (2018) 2018:1465348. doi: 10.1155/2018/1465348 29780815 PMC5892233

[39] Andersen CL Boisen MM Sikora MJ Ma T Tseng G Suryawanshi S. The evolution of estrogen receptor signaling in the progression of endometriosis to endometriosis-associated ovarian cancer. Horm Cancer. (2018) 9:399–407. doi: 10.1007/s12672-018-0350-9 30302736 PMC10355926

[40] Wang D Guo C Li Y Zhou M Wang H Liu J. Oestrogen up-regulates DNMT1 and leads to the hypermethylation of RUNX3 in the Malignant transformation of ovarian endometriosis. Reprod BioMed Online. (2022) 44:27–37. doi: 10.1016/j.rbmo.2021.06.030 34799276

[41] Mortlock S Corona RI Kho PF Pharoah P Seo JH Freedman ML. A multi-level investigation of the genetic relationship between endometriosis and ovarian cancer histotypes. Cell Rep Med. (2022) 3:100542. doi: 10.1016/j.xcrm.2022.100542 35492879 PMC9040176

[42] Bulun SE Wan Y Matei D. Epithelial mutations in endometriosis: link to ovarian cancer. Endocrinology. (2019) 160:626–38. doi: 10.1210/en.2018-00794 PMC6382454 30657901

[43] El Hout M Dos Santos L Hamaï A Mehrpour M. A promising new approach to cancer therapy: Targeting iron metabolism in cancer stem cells. Semin Cancer Biol. (2018) 53:125–38. doi: 10.1016/j.semcancer.2018.07.009 30071257

[44] Toyokuni S. Role of iron in carcinogenesis: cancer as a ferrotoxic disease. Cancer Sci. (2009) 100:9–16. doi: 10.1111/j.1349-7006.2008.01001.x 19018762 PMC11158384

[45] Nagababu E Rifkind JM. Heme degradation by reactive oxygen species. Antioxid Redox Signal. (2004) 6:967–78. doi: 10.1089/ars.2004.6.967 15548894

[46] Kobayashi H. Potential scenarios leading to ovarian cancer arising from endometriosis. Redox Rep. (2016) 21:119–26. doi: 10.1179/1351000215Y.0000000038 PMC6837701 26317761

[47] Scutiero G Iannone P Bernardi G Bonaccorsi G Spadaro S Volta CA. Oxidative stress and endometriosis: A systematic review of the literature. Oxid Med Cell Longev. (2017) 2017:7265238. doi: 10.1155/2017/7265238 29057034 PMC5625949

[48] Johannes L Jacob R Leffler H. Galectins at a glance. J Cell Sci. (2018). doi: 10.1242/jcs.208884 29717004

[49] Hisrich BV Young RB Sansone AM Bowens Z Green LJ Lessey BA. Role of human galectins in inflammation and cancers associated with endometriosis. Biomolecules. (2020) 10:230. doi: 10.3390/biom10020230 32033052 PMC7072718

[50] Su KM Wang PH Yu MH Chang CM Chang CC. The recent progress and therapy in endometriosis-associated ovarian cancer. J Chin Med Assoc. (2020) 83:227–32. doi: 10.1097/JCMA.0000000000000262 PMC13048167 31985569

[51] Leenen S Hermens M de Vos van Steenwijk PJ Bekkers RLM Van Esch EMG. Immunologic factors involved in the Malignant transformation of endometriosis to endometriosis-associated ovarian carcinoma. Cancer Immunol Immunother. (2021) 70:1821–9. doi: 10.1007/s00262-020-02831-1 PMC10992190 33411080

[52] Zanetta GM Webb MJ Li H Keeney GL. Hyperestrogenism: a relevant risk factor for the development of cancer from endometriosis. Gynecol Oncol. (2000) 79:18–22. doi: 10.1006/gyno.2000.5905 11006024

[53] Yarmolinsky J Relton CL Lophatananon A Muir K Menon U Gentry-Mahara A. Appraising the role of previously reported risk factors in epithelial ovarian cancer risk: A Mendelian randomization analysis. PloS Med. (2019) 16:e1002893. doi: 10.1371/journal.pmed.1002893 31390370 PMC6685606

[54] Lee HJ Lee B Choi H Kim T Kim Y Kim YB. Impact of hormone replacement therapy on risk of ovarian cancer in postmenopausal women with *de novo* endometriosis or a history of endometriosis. Cancers (Basel). (2023) 15:1708. doi: 10.3390/cancers15061708 36980597 PMC10046182

[55] Udomsinkul P Triratanachart S Oranratanaphan S. Risk factors for endometriotic-cyst associated ovarian cancer: A case controlled study. Taiwan J Obstet Gynecol. (2020) 59:269–74. doi: 10.1016/j.tjog.2020.01.016 32127149

[56] Giannella L Marconi C Di Giuseppe J Delli Carpini G Fichera M Grelloni C. Malignant transformation of postmenopausal endometriosis: A systematic review of the literature. Cancers (Basel). (2021) 13:4026. doi: 10.3390/cancers13164026 34439184 PMC8394809

[57] Li Q Sun Y Zhang X Wang L Wu W Wu M. Endometriosis-associated ovarian cancer is a single entity with distinct clinicopathological characteristics. Cancer Biol Ther. (2019) 20:1029–34. doi: 10.1080/15384047.2019.1595278 PMC6605994 30913953

[58] Zhou Y Hua KQ. Ovarian endometriosis: risk factor analysis and prediction of Malignant transformation. Prz Menopauzalny. (2018) 17:43–8. doi: 10.5114/pm.2018.74902 PMC5925200 29725285

[59] Murakami K Kotani Y Shiro R Takaya H Nakai H Matsumura N. Endometriosis-associated ovarian cancer occurs early during follow-up of endometrial cysts. Int J Clin Oncol. (2020) 25:51–8. doi: 10.1007/s10147-019-01536-5 31473885

[60] Harris HR Peres LC Johnson CE Guertin KA Beeghly A Bandera EV. Racial differences in the association of endometriosis and uterine leiomyomas with the risk of ovarian cancer. Obstet Gynecol. (2023) 141:1124–38. doi: 10.1097/AOG.0000000000005191 PMC10440275 37159277

[61] Khoja L Weber RP. Australian Ovarian Cancer Study Group, et al. Endometriosis and menopausal hormone therapy impact the hysterectomy-ovarian cancer association. Gynecol Oncol. (2022) 164:195–201. doi: 10.1016/j.ygyno.2021.10.088 34776242 PMC9444325

[62] Ring LL Baandrup L Zheng G Gottschau M Dehlendorff C Mellemkjær L. Hysterectomy and risk of epithelial ovarian cancer by histologic type, endometriosis, and menopausal hormone therapy. Cancer Epidemiol. (2023) 84:102359. doi: 10.1016/j.canep.2023.102359 37054550

[63] Chiang AJ Chang C Huang CH Huang WC Kan YY Chen J. Risk factors in progression from endometriosis to ovarian cancer: a cohort study based on medical insurance data. J Gynecol Oncol. (2018) 29:e28. doi: 10.3802/jgo.2018.29.e28 29400021 PMC5920215

[64] Wang S Lang J. Current research status and prospects of ovarian cancer related to endometriosis. Chin J Org Chem. (2017) 33:360–4. doi: 10.19538/j.fk2017040109

[65] Wang J Qu Q Zhang S. Expert consensus on the diagnosis and treatment of ovarian cancer related to endometriosis in shandong. Shandong Med J. (2022) 62:1–6.

[66] Shinmura H Yoneyama K Harigane E Tsunoda Y Fukami T Matsushima T. Use of tumor markers to distinguish endometriosis-related ovarian neoplasms from ovarian endometrioma. Int J Gynecol Cancer. (2020) 30:831–6. doi: 10.1136/ijgc-2020-001210 PMC7362875 32354795

[67] Magalhães JS Jammal MP Crispim PCA Murta EFC Nomelini RS. Role of biomarkers CA-125, CA-15.3 and CA-19.9 in the distinction between endometriomas and ovarian neoplasms. Biomarkers. (2021) 26:268–74. doi: 10.1080/1354750X.2021.1885490 33554683

[68] Xu T Zhang L Jiang Y Wan Y Zhou S Sun G. Clinical characteristics and serum CA19-9 combined with HE4 are valuable in diagnosing endometriosis-associated ovarian cancer. Int J Gynaecol Obstet. (2023) 162:472–8. doi: 10.1002/ijgo.14695 36708088

[69] Shin KH Kim HH Kwon BS Suh DS Joo JK Kim KH. Clinical usefulness of cancer antigen (C) 125, human epididymis 4, and CA72-4 levels and risk of ovarian Malignancy algorithm values for diagnosing ovarian tumors in korean patients with and without endometriosis. Ann Lab Med. (2020) 40:40–7. doi: 10.3343/alm.2020.40.1.40 PMC6713655 31432638

[70] Dochez V Caillon H Vaucel E Dimet J Winer N Ducarme G. Biomarkers and algorithms for diagnosis of ovarian cancer: CA125, HE4, RMI and ROMA, a review. J Ovarian Res. (2019) 12:28. doi: 10.1186/s13048-019-0503-7 30917847 PMC6436208

[71] Testa AC Timmerman D Van Holsbeke C Zannoni GF Fransis S Moerman P. Ovarian cancer arising in endometrioid cysts: ultrasound findings. Ultrasound Obstet Gynecol. (2011) 38:99–106. doi: 10.1002/uog.8970 21351179

[72] Huang KJ Li YX Wu CJ Chang WC Wei LH Sheu BC. Sonographic features differentiating early-stage ovarian clear cell carcinoma from endometrioma with atypical features. J Ovarian Res. (2022) 15:84. doi: 10.1186/s13048-022-01019-8 35836288 PMC9284754

[73] Moro F Magoga G Pasciuto T Mascilini F Moruzzi MC Fischerova D. Imaging in gynecological disease (13): clinical and ultrasound characteristics of endometrioid ovarian cancer. Ultrasound Obstet Gynecol. (2018) 52:535–43. doi: 10.1002/uog.19026 29418038

[74] Michielsen K Dresen R Vanslembrouck R Keyzer FD Vandecaveye V. Diagnostic value of whole body diffusion-weighted MRI compared to computed tomography for pre-operative assessment of patients suspected for ovarian cancer. Eur J Cancer. (2017) 83:88–98. doi: 10.1016/j.ejca.2017.06.010 28734146

[75] Yoshimoto C Takahama J Iwabuchi T Uchikoshi M Shigetomi H Kobayashi H. Transverse relaxation rate of cyst fluid can predict Malignant transformation of ovarian endometriosis. Magn Reson Med Sci. (2017) 16:137–45. doi: 10.2463/mrms.mp.2016-0028 PMC5600073 27646154

[76] Zhang X Li M Tang Z Li X Song T. Differentiation between endometriosis-associated ovarian cancers and non- endometriosis-associated ovarian cancers based on magnetic resonance imaging. Br J Radiol. (2021) 94:20201441. doi: 10.1259/bjr.20201441 33882252 PMC9327745

[77] Veneziani AC Gonzalez-Ochoa E Alqaisi H Madariaga A Bhat G Rouzbahman M. Heterogeneity and treatment landscape of ovarian carcinoma. Nat Rev Clin Oncol. (2023) 20:820–42. doi: 10.1038/s41571-023-00819-1 37783747

[78] Kitamura S Yamaguchi K Murakami R Furutake Y Higasa K Abiko K. PDK2 leads to cisplatin resistance through suppression of mitochondrial function in ovarian clear cell carcinoma. Cancer Sci. (2021) 112:4627–40. doi: 10.1111/cas.15125 PMC8586679 34464482

[79] Itamochi H Kigawa J Akeshima R Sato S Kamazawa S Takahashi M. Mechanisms of cisplatin resistance in clear cell carcinoma of the ovary. Oncology. (2002) 62:349–53. doi: 10.1159/000065067 12138243

[80] Bizzarri N Imterat M Fruscio R Giannarelli D Perrone AM Mancari R. Lymph node staging in grade 1-2 endometrioid ovarian carcinoma apparently confined to the ovary: Is it worth? Eur J Cancer. (2023) 195:113398. doi: 10.1016/j.ejca.2023.113398 37890354

[81] Deng T Liu K Chen L Chen X Li HW Guo H. A prospective randomized multicenter trial for lymphadenectomy in early-stage ovarian cancer: LOVE study. J Gynecol Oncol. (2023) 34:e52. doi: 10.3802/jgo.2023.34.e52 37116952 PMC10157337

[82] Harter P Sehouli J Lorusso D Reuss A Vergote I Marth C. A randomized trial of lymphadenectomy in patients with advanced ovarian neoplasms. N Engl J Med. (2019) 380:822–32. doi: 10.1056/NEJMoa1808424 30811909

[83] Van Driel WJ Koole SN Sikorska K Schagen van Leeuwen JH Schreuder HW Hermans RH. Hyperthermic intraperitoneal chemotherapy in ovarian cancer. N Engl J Med. (2018) 378:230–40. doi: 10.1056/NEJMoa1708618 29342393

[84] Aronson SL Lopez-Yurda M Koole SN van Leeuwen JHS Schreuder HW Hermans RH. Cytoreductive surgery with or without hyperthermic intraperitoneal chemotherapy in patients with advanced ovarian cancer (OVHIPEC-1): final survival analysis of a randomised, controlled, phase 3 trial. Lancet Oncol. (2023) 24:1109–18. doi: 10.1016/S1470-2045(23)00396-0 37708912

[85] Koole S van Stein R Sikorska K Barton D Perrin L Brennan D. Primary cytoreductive surgery with or without hyperthermic intraperitoneal chemotherapy (HIPEC) for FIGO stage III epithelial ovarian cancer: OVHIPEC-2, a phase III randomized clinical trial. Int J Gynecol Cancer. (2020) 30:888–92. doi: 10.1136/ijgc-2020-001231 PMC8202725 32205449

[86] Villarejo Campos P Sánchez García S Amo-Salas M Amo-Salas M García Santos E López de la Manzanara C. Paclitaxel as HIPEC-drug after surgical cytoreduction for ovarian peritoneal metastases: A randomized phase III clinical trial (HIPECOV). Curr Oncol. (2024) 31:660–71. doi: 10.3390/curroncol31020048 PMC10888026 38392042

[87] Clamp AR James EC McNeish IA Dean A Kim JW O'Donnell DM. Weekly dose-dense chemotherapy in first-line epithelial ovarian, fallopian tube, or primary peritoneal cancer treatment (ICON8): overall survival results from an open-label, randomised, controlled, phase 3 trial. Lancet Oncol. (2022) 23:919–30. doi: 10.1016/S1470-2045(22)00283-2 PMC9630160 35690073

[88] Tattersall A Ryan N Wiggans AJ Rogozińska E Morrison J. Poly(DP-ribose) polymerase (PARP) inhibitors for the treatment of ovarian cancer. Cochrane Database Syst Rev. (2022) 2:CD007929. doi: 10.1002/14651858.CD007929.pub4 35170751 PMC8848772

[89] O'Malley DM Matulonis UA Birrer MJ Castro CM Gilbert L Vergote I. Phase Ib study of mirvetuximab soravtansine, a folate receptor alpha (FRα)-targeting antibody-drug conjugate (DC), in combination with bevacizumab in patients with platinum-resistant ovarian cancer. Gynecol Oncol. (2020) 157:379–85. doi: 10.1016/j.ygyno.2020.01.037 32081463

[90] Matulonis UA Lorusso D Oaknin A Pignata S Denys H Colombo N. Efficacy and safety of mirvetuximab soravtansine in patients with platinum-resistant ovarian cancer with high folate receptor alpha expression: results from the SORAYA study. J Clin Oncol. (2023) 41:2436–45. doi: 10.1200/JCO.22.01900 PMC10150846 36716407

[91] Moore KN Bookman M Sehouli J Miller A Anderson C Scambia G. Atezolizumab, bevacizumab, and chemotherapy for newly diagnosed stage III or IV ovarian cancer: placebo-controlled randomized phase III trial (IMagyn050/GOG 3015/ENGOT-OV39). J Clin Oncol. (2021) 39:1842–55. doi: 10.1200/JCO.21.00306 PMC8189598 33891472

[92] Monk BJ Colombo N Oza AM Fujiwara K Birrer MJ Randall L. Chemotherapy with or without avelumab followed by avelumab maintenance versus chemotherapy alone in patients with previously untreated epithelial ovarian cancer (JAVELIN Ovarian 100): an open-label, randomised, phase 3 trial. Lancet Oncol. (2021) 22:1275–89. doi: 10.1016/S1470-2045(21)00342-9 34363762

[93] Ono M Miyamoto T Asaka R Uchikawa J Ando H Tanaka Y. Establishment of a novel model of endometriosis-associated ovarian cancer by transplanting uterine tissue from Arid1a/Pten knockout mice. Sci Rep. (2023) 13:8348. doi: 10.1038/s41598-023-35292-4 37221199 PMC10205720

